# A Case of Congenital Isolated Adrenocorticotropic Hormone Deficiency Caused by Two Novel Mutations in the *TBX19* Gene

**DOI:** 10.3389/fendo.2019.00251

**Published:** 2019-04-18

**Authors:** Kong Weijing, Zou Liping, Zhang Tiantian, Zhang Pei, Meng Yan

**Affiliations:** ^1^Department of Pediatrics, Chinese PLA General Hospital, Beijing, China; ^2^Department of Pediatrics, Beijing Friendship Hospital, Capital Medical University, Beijing, China; ^3^Department of Pediatrics, Xuanwu Hospital Capital Medical University, Beijing, China

**Keywords:** adrenocorticotropic hormone, cortisol, thyroid-stimulating hormone, special appearance, *TBX19* gene

## Abstract

Congenital isolated adrenocorticotropic hormone (ACTH) deficiency (CIAD) is a rare disorder which can result in 20% mortality in the neonatal period if misdiagnosed. A 2 years and 7 months old boy was hospitalized many times because of recurrent hypoglycemia. On initial physical examination, the patient showed special appearance and indications of fast growth (≥P97). Laboratory investigations revealed low levels of ACTH and cortisol in his plasma. Except thyroid-stimulating hormone, the anterior pituitary hormone concentrations were normal. Molecular data showed compound heterozygosity for two novel mutations in the *TBX19* gene (encoding the transcription factor T-Box 19). Mutation c.205C>T was inherited from mother and the fragment deletion (from g.168,247,374 to g.168,278,264) was from father. Hydrocortisone replacement therapy was effective. We reported two novel *TBX19* mutations, expanding the mutation spectrum of this disorder, in a CIAD patient who presented with special appearance, signs of fast growth, and thyroid-stimulating hormone derangement. In addition, for avoiding misdiagnosis, criterion for ACTH and cortisol detection of CIAD should be established.

## Background

Congenital isolated adrenocorticotropic hormone (ACTH) deficiency (CIAD) (MIM 201400) is a rare disorder, characterized by severe hypoglycemia, which is associated with seizures in about 50% of cases. Endocrine profile of CIAD patients is characterized by very low plasma ACTH levels and extremely low plasma cortisol levels. The low plasma cortisol levels did not response to either acute corticotropin-releasing hormone (CRH) or ACTH, but may respond to repeated ACTH stimulation (1). CIAD can result in 20% mortality in the neonatal period if unrecognized (2). CIAD is an autosomal recessive inherited disease that is caused by homozygous or compound heterozygous mutations in the *TBX19* (MIM 604614) (3). The protein of *TBX19*, located on chromosome 1q24, is a member of T-box family, which has crucial roles throughout development (4). In the present study, we report a CIAD case presenting with recurrent seizure and hypoglycemia caused by two novel TBX19 mutations. There are 27 mutations of TBX19 were included in the Human Genome Mutation Database (HGMD; www.hgmd.cf.ac.uk), so two mutations in our case would add almost 10% to the know mutations.

## Case Report

A 2 years and 7 months old boy was referred to our hospital with recurrent hypoglycemia and seizure for more than 2 years.

The infant was G1P1, a full-term baby, with a birth weight of 3.6 kg. The neonate experienced two episodes of hypoglycemic convulsions on days 5 and 13 and was therefore admitted to the neonatal intensive care unit (ICU). From 6 months old to 28 months old, the infant was hospitalized many times because of recurrent hypoglycemia (his blood glucose value range 0.1–1.3 mmol/L) and convulsions, which occurred because of fever, low calorific intake and diarrhea.

At the age of 2 years and 7 months, the patient was referred to our hospital because of recurrent hypoglycemic convulsions for more than 2 years. On initial physical examination, the patient weighed 16.6 kg (≥P97) and was 103 cm long (≥P97). He showed decreased activity and weakness, with special appearance (hypertelorism, narrow palpebral fissures, epicanthus, low-set ears, auricular malformation, and transverse palmar crease in right hand). The boy had normal IQ. No disturbances in the neuro-psycho-motor development. Jaundice appeared on the fourth day after birth. Total bilirubin (316.6 μmol/L) was tested on the 6th day, indirect bilirubin (303.9 μmol/L) was dominate. Jaundice gradually declined after intermittent phototherapy and completely disappeared on day 19. No signs of liver disease.

Laboratory investigations revealed low plasma cortisol and ACTH concentrations. There was no obvious circadian rhythm of ACTH and cortisol levels. An ACTH test was failed to stimulate the production of cortisol. The thyroid function tests, kidney function, and electrolytes were all normal. Except thyroid-stimulating hormone (TSH), the anterior pituitary hormone concentrations were normal (Table 1). The blood sugar was normal under non-stress condition with continuous glucose monitoring system (range 4.7 ± 0.6 mmol/L). Based on the profile of the hormonal and clinical characteristics, CIAD was suspected.

**Table 1:** Clinical findings and laboratory results.

	**On admission**	**Final examination**
Age	2 years and 7 months	3 years and 6 months
Weight (kg)	16.6	17.9
Height (cm)	103	107
BMI (kg/m^2^)	15.6	15.6
**LABORATORY TESTS (REFERENCE RANGES)**		
FBG (3.4–6.1 mmol/L)	3.77	4.68
Na (130–150 mmol/L)	140.7	140.3
K (3.5–5.5 mmol/L)	4.11	4.36
ACTH (1.6–13.9 pmol/L)	0 a.m.: 4.82 8 a.m.: 4.13 4 p.m.: 4.88	0 a.m.: 4.71 8 a.m.: 4.93 4 p.m.: 5.28
Cortisol (193.2–690 nmol/L)	**0 a.m.:** ** <25.7** **8 a.m.:** ** <25.7** **4 p.m.:** ** <25.7**	0 a.m.: 277.32 8 a.m.: 981.63 4 p.m.: 418.22
TSH (0.35–5.5 mU/L)	**9.54**	1.54
FT4 (10.42–24.32 pmol/L)	17.17	15.02
GH (0.06–5 ug/L)	2.85	0.711
DHEAS (80–560 ug/dl)	<15	
17α-OHP (ng/ml)	<0.1	
Karyotype	Normal	
Bone age	Normal	
Adrenal USG	Normal	
Cranial and pituitary MRI	Normal	

*ACTH, adrenocorticotropic hormone; BMI, body mass index; DHEAS, dehydroepiandrosterone sulfate; FBG, fasting blood glucose; FT4, free thyroxine; GH, growth hormone; K, potassium; Na, sodium; MRI, magnetic resonance imaging; PRL, prolactin; TSH, thyroid-stimulating hormone; UCP, Urinary free cortisol; USG, ultrasonography; 17α-OHP, 17α-hydroxyprogesterone (abnormal findings are shown in bold)*.

**Figure 1:**
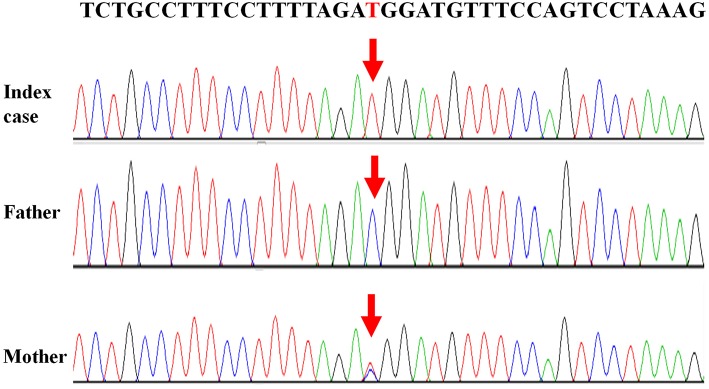
Mutations of “*TBX19* gene” detected in family members.

Written informed consent was obtained from the patient's legal guardians to perform genetic analysis, publish the manuscript and fully explain the purpose and nature of all the procedures used. Data of next generation sequencing showed two novel heterozygous variations (c.205C>T (p.R69W) in exon 2 and a large fragment deletion). Molecular analysis revealed that c.205C>T was inherited from mother and fragment deletion (from g.168,247,374 to g.168,278,264) was inherited from father. c.205C>T was confirmed by Sanger sequencing (Figure 1). The fragment deletion (from g.168,247,374 to g.168,278,264), including exon 2 to exon 8, was confirmed using quantitative real-time (qPCR) (Table 2). Karyotype and gene copy number variation screening were normal.

**Table 2:** Fragment deletion confirmed by qPCR.

	**Index case**	**Father**	**Mother**
Exon2	0.399	0.486	1.078
Exon5	0.557	0.572	1.031
Exon8	0.546	0.582	0.861

*The data is 2^−ΔΔCt^. 2^−ΔΔCt^ between 0.7 and 1.3 is in the normal range*.

The patient was treated with hydrocortisone supplementation with total daily dose of 15 mg/m^2^.d given three times a day. He was discharged home and with treatment of hydrocortisone replacement therapy for life-span. On the final examination, his weight was 17.9 kg (P90-P97) and length was 107 cm (P90–P97). After treatment with hydrocortisone over 1 year and 8 months, the height of boy is less than P75. Insulin-like growth factor 1 (IGF1) was measured (153 ng/mL), which is in the normal range (50–286 ng/mL). Although his level of cortisol was up-regulated to the normal level and showed a regular circadian rhythm, there was still no obvious circadian rhythm of ACTH. Without treatment with thyroid hormone, increased levels of TSH also returned to the reference range (Table 1). Compared with the first initial examination, the infant had more energy for normal activities.

The patient has continued to follow-up with the pediatric clinic and continues to show significant improvement, i.e., no more seizures or hypoglycemic episodes have occurred. He is now under the care of his local pediatricians.

## Discussion

ACTH is produced from the precursor polypeptide proopiomelanocortin (POMC) by the enzymes proconvertase-1 and proconvertase-2; therefore, any factors inducing deregulated production or abnormal of POMC protein will induce ACTH insufficiency (5). TBX19 can bind to the *POMC* promoter to activate transcription (6). Mutations in *TBX19*, which can disrupt TBX19 function, can cause CIAD. In the present study, we reported a CIAD case with two novel mutations of *TBX19* who presented with recurrent hypoglycemia and seizures.

The causes of hypoglycemic convulsions in infants are complicated. Children under 5 years old with repeated episodes of severe hypoglycemia may develop permanent neurological damage. It is important to determine the underlying cause of hypoglycemia. Early diagnosis allows earlier intervention to improve the chance of targeted treatment. In the present case, the patient developed hypoglycemic episodes in the neonatal period. The cortisol level in his plasma was significantly lower and was not stimulated by ACTH. He also has TSH derangement and signs of fast growth. However, other pituitary hormone levels, adrenal ultrasound and pituitary magnetic resonance imaging results were normal. We also noticed his special appearance, which, to the best of our knowledge, has never been reported in infant CIAD. However, there were no gene mutations that were directly related to his special appearance. Based on the genetic analysis, clinical features, and the outcome of treatment, the patient could be diagnosed as CIAD.

Several hormonal abnormalities have been reported in cases of isolated ACTH deficiency (7–9). The GH value in Table 1 was within the reference range, because the value was tested before stimulation. We could not conduct a growth hormone stimulation test, because it could induce hypoglycemia which is very dangerous for the patient. After treatment with hydrocortisone, speed of growth got slowly, which indicates the relationship between cortisol and GH. Normal level of IGF1 after treatment also indicates that supplement of hydrocortisone can reduce the GH level and reduce the speed of growth of CIAD.

Reference values for routine laboratory testing of ACTH and cortisol are about 1.6–13.9 pmol/L and 193.2–690 nmol/L, respectively (3, 10, 11). In three recent cases, ACTH and cortisol levels in the plasma were very low, which were lower than lower limit of the reference value and perfectly consistent with routine laboratory test (3, 11). In our case, the plasma level of cortisol was <25.7 nmol/L, which is less than the lower limit of reference value. Level of ACTH in plasma was 4.13 pmol/L, which is within the normal range for the regular test but was abnormal in CIAD based on the classification of Couture et al. Couture et al. reported 91 IAD patients to better characterize the phenotype and the genotype of CIAD, which was the largest neonatal CIAD case series reported to date. The patients were identified as belonging to three distinct groups: neonatal onset complete, partial IAD, or late onset IAD, with or without mutations of *TBX19*. Plasma levels of ACTH and cortisol in complete ACTH deficiency patients were 6.9 ± 3.5 pmol/L and 28 ± 10 nmol/L, respectively (12). ACTH level in plasma of our patient is 4.13 pmol/L, which is in the range of 6.9 ± 3.5 pmol/L. According to the research of Couture et al., our patient could be identified as neonatal onset complete IAD.

Plasma level of ACTH in our patients was at a low level according to reference value, but it was not less than lower limit of reference value. The possible explanation may be: TBX19 is a transcriptional activator of POMC; however, it is not the only transcriptional regulators of POMC. There are many other transcriptional regulators of POMC gene, such as bHLH, Smad, and Stat (13). In our patient, mutations in TBX19 may influence the production of ACTH, but other regulators unknown may reduce the influence of TBX19. It is a complex mechanism, but for now, criterion of detection of ACTH and cortisol for CIAD patients should be established to avoid misdiagnosis.

Couture et al. reported some sporadic clinical anomalies in CIAD patients, such as mental retardation, congenital cardiomyopathy, Arnold-Chiari type I malformation, triple X syndrome, mild dystrophic features, and unilateral choana atresia (12). These sporadic clinical anomalies are hardly explained by mutations of *TBX19*. In our case, there are no mental retardation, cardiac-related diseases, choana atresia, and no signs of Arnold-Chiari type I malformation. Karyotype and gene copy number variation screening were normal, so triple X syndrome was ruled out. Most of time, nutrition supply of this boy was normal, even a little excessive, so there were no dystrophic features. The clinical anomalies of our patient are not included in the cohorts which were reported by Couture et al. and expand the spectrum of clinical anomalies in CIAD.

Currently, there are 27 TBX19 mutations in HGMD. In our cases, we reported two novel mutations which were never reported. c.205C>T (p.R69W), a missense mutation, may disrupt DNA binding, since this residue is inside the T-box region. Amino acids from 45 to 218 of TBX19 formed T-box which is essential for DNA binding (14). Next generation sequencing revealed a large fragment deletion, and Sanger sequence showed that the c.205C>T mutation was homozygous mutation (Figure 1). qPCR (Table 2) confirmed that the large fragment deletion was from g.168,247,374 to g.168,278,264. Deletion of this fragment also disrupted structure and function of TBX19. TBX19 can bind to the *POMC* promoter and stimulate transcription of *POMC* (6). If the function of TBX19 was disrupted or destroyed, transcription of *POMC* would be reduced, which induced the production of ACTH. Although phenotypes of CIAD have relationship with TBX19, the pathogenesis of CIAD was not so clear. More case reports and clinical research are required to better characterize the phenotype and the genotype of this rare disease.

## Concluding Remarks

In conclusion, we reported a CIAD case with two novel mutations that presented with recurrent seizures and hypoglycemia. When an infant shows recurrent hypoglycemia, with seizures and low plasma levels of ACTH and cortisol, CIAD should be take into consideration, even if the patient has other hormonal abnormalities. Genetic analysis is a powerful tool to confirm the diagnosis, which helped us to identify two novel mutations in our patient. In addition, the lack of an obvious circadian rhythm of ACTH and cortisol before treatment may be a specific characteristic of this disease.

## Ethics Statement

This study was carried out in accordance with the recommendations of Declaration of Helsinki, ethics committee of Chinese PLA General Hospital with written informed consent from all subjects. All subjects gave written informed consent in accordance with the Declaration of Helsinki. The protocol was approved by the ethics committee of Chinese PLA General Hospital.

## Author Contributions

ZT and ZP managed and followed up the case. KW collected the data and wrote the manuscript. ZL supervised the management and follow up of the case. MY supervised the management and follow up of the case, and the writing of the article. All the authors revised and approved the final manuscript and agreed to be accountable for the content of the work.

### Conflict of Interest Statement

The authors declare that the research was conducted in the absence of any commercial or financial relationships that could be construed as a potential conflict of interest.
